# Food Addiction, Saturated Fat Intake, and Body Mass Index in Peruvian Adults: A Cross-Sectional Survey

**DOI:** 10.1155/2021/9964143

**Published:** 2021-07-21

**Authors:** Dulce E. Lopez-Lopez, Ivett K. Saavedra-Roman, Yaquelin E. Calizaya-Milla, Jacksaint Saintila

**Affiliations:** Escuela Profesional de Nutrición Humana, Facultad de Ciencias de la Salud, Universidad Peruana Unión, Lima, Peru

## Abstract

**Background:**

Cardiovascular diseases (CVDs) constitute one of the main public health problems and represent a greater risk of mortality and morbidity for the world population. The objective of the study was to determine food addiction, saturated fat intake, and body mass index (BMI) in Peruvian adults.

**Materials and Methods:**

A cross-sectional online survey was applied to 394 Peruvian adults over 18 years old residing in the three regions of the country. Participant data was collected through a prestructured online electronic survey. Food addiction was assessed using the Yale Food Addiction Scale self-administered questionnaire. A validated food frequency questionnaire was used to measure saturated fat intake. Finally, the sociodemographic and anthropometric variables were collected through a registration form.

**Results:**

There were no significant differences in food addiction between men and women (*p* < 0.05). More than half of the participants who presented food addiction are overweight (54.1%, *p* < 0.001). The highest proportion of those who had a high intake of saturated fat had a food addiction (62.6%, *p* < 0.001). The highest percentage of men who were overweight was higher compared to women (49.7% vs. 38.4%, *p* < 0.05).

**Conclusion:**

The findings of this study suggest that addictive eating behaviors and high saturated fat intake should be considered as part of efforts to prevent problems related to eating, obesity, and CVD.

## 1. Introduction

Cardiovascular diseases (CVDs) constitute one of the main public health problems and represent an increased risk of mortality and morbidity for the world population [1]. The prevalence of CVD is steadily increasing in both developing and developed countries [2]. According to available evidence, in 2012, 17 million deaths related to these pathologies were reported. In fact, they represent almost a third of the deaths that occur worldwide [3]. Peru is not far from this reality. CVDs are the leading cause of death [4]. A report published in 2016 estimated that 16% of the Peruvian population over 20 years of age suffers from CVD and more than 2,000 Peruvians die from a type of heart failure [5].

Among the most important risk factors for CVD are high blood pressure, BMI, and high cholesterol and saturated fat intake, which are conditioned by inappropriate eating habits [6, 7]. In fact, the consumption of a diet based on meat and with a higher content of cholesterol, saturated and trans fat can increase serum cholesterol concentrations leading to an increased risk of CVD [8]. Faced with this situation, the Peruvian state approved the Manual of Advertising Warnings within the framework of what is established in Law N^o^. 30021, Law for the Promotion of Healthy Eating, whose objective is to inform the population about the nutritional content of processed and ultra-processed foods to reduce diseases linked to overweight, obesity, and CVD [9]. Faced with this scenario, the national food production industry was forced to put a nutritional warning front label on processed foods with a high content of sugar, sodium, and saturated fats and to eliminate the content of trans fats in products.

On the other hand, the concept of food addiction has always existed in popular culture [10], even though there is still no universally accepted definition [11]. However, it was not until 2009 that researchers first validated an instrument to measure food addiction in people [12]; since then, more than fifty peer-reviewed articles have examined the prevalence of food addiction [10]. The prevalence of food addiction is estimated to vary between 5 and 10% of the general population [13, 14]. Food addiction is different from other eating disorders like anorexia or bulimia [15]. However, it is positively associated with binge eating, depression [15], food craving, and impulsiveness [16]. People with food addiction report higher dietary fat intake than those without food addiction [11, 17]. In addition, it is suggested that foods high in fat, salt, and sugar may be more appetizing and more attractive to some people [18], and they could be related to overeating and chronic noncommunicable diseases such as obesity [14] and CVD [19]. Significant changes in lifestyle, especially in eating habits with healthier food choices, can play an important role in the prevention of obesity and associated chronic diseases [20].

Prevention of CVD can be achieved through early identification and encouragement of a healthy lifestyle that includes regular physical activity, healthy dietary patterns such as the Mediterranean diet [21], and a plant-based diet with an emphasis on minimally processed foods and naturals such as fruits, vegetables, nuts, whole grains, and seeds and lower consumption of ultra-processed foods rich in saturated and trans fats [22, 23]. In the same way, other healthy foods such as fish and fermented or low-fat dairy products can be beneficial in preventing CVD [21]. International organizations such as the American Heart Association recommend that saturated fat consumption for healthy adults be limited to less than 7% of total daily calories, trans fat to less than 1% of total daily calories, and cholesterol to less than 300 mg daily [24]. Similarly, since March 2021, the Peruvian state requires that no processed and ultra-processed food products contain trans fats such as cream, butter, beef fat (tallow), chicken fat, pork fat (lard), margarine in the bar, and shortening [9].

Despite the available evidence, there are still important gaps in the literature on food addiction [13]. Studies have generally examined food addiction in targeted populations or clinical samples with eating disorders such as binge eating disorder and obesity [15]. However, the existing literature has largely not addressed one of the fundamental aspects of food addiction: its relationship with BMI and the specific intake of nutrients such as saturated fats, which do not fulfill an important biological function for the organism. Therefore, the objective of the study was to evaluate food addiction, saturated fat intake, and BMI in Peruvian adults.

## 2. Materials and Methods

### 2.1. Research Design and Participants

A cross-sectional online survey was applied to 394 adults over 18 years of age. The survey included questions related to sociodemographic and anthropometric aspects, fat intake, and food addiction. The participants were recruited during the beginning of February and March 2021 through e-mail, WhatsApp Messenger, Instagram, and Facebook Messenger. The survey was available on SurveyMonkey Pro (SurveyMonkey.com, LLC, Palo Alto, CA) and contained a description of the objective of the study and the purposes of data collection, the rights of the participants, and the confidentiality in the use of the data. Participants were not offered any incentives to participate in the study. In addition, they were informed that they could withdraw from the study at any time if they wanted to. Finally, it was explained to them that they could express their desire to participate in the study by marking the option “I wish to participate” and if not, they were free to mark the option “I do not wish to participate” and went to the closing page of the questionnaire. The study was carried out in harmony with the Helsinki Declaration, and the study protocol was approved by the Bioethical Research Committee of the Universidad Peruana Unión and registered under number 042–2021/UPeU/FCS. A total of 418 people participated in the study; however, 24 participants were excluded for not having completed the questionnaire correctly and for being a minor.

### 2.2. Data Collection Instruments

#### 2.2.1. Sociodemographic and Anthropometric Data

A registration form prepared by the study researchers was used. It is composed of 6 questions, considering the following dimensions: sociodemographic profile (sex, place of origin, and level of education), lifestyle (hours of sleep, physical activity, and the number of meals per day), and dietary patterns (vegetarian and nonvegetarian).

#### 2.2.2. Food Addiction Questionnaire

To determine whether or not there is a food addiction, the self-administered Yale Food Addiction Scale instrument created in 2009 was used [12]. And adapted to the Spanish language, it obtained a Cronbach's Alpha of 0.80 [25]. The instrument is composed of 25 items divided into five components that operationalize the manifestation of the criteria proposed by the DSM-5 to identify food addiction and its dependence on substances. The scale includes dichotomous response options in which yes, it is equal to 0, and no, it is equal to 1 point. In addition, it contains frequency response options: never = 0 points, once a month = 1 point, 2–4 times a month = 2 points, 2–3 times a week = 3 points, and 4 or more times a week/daily = 4 points. The sum of the final results indicates that there is addiction to food if at least three of the criteria that make up the scale are positive.

#### 2.2.3. Fat Consumption Questionnaire

To assess the participants' habitual fat intake, a food frequency questionnaire created and validated in a previous study was used [26]. However, some equivalent elements were included to facilitate understanding of the questionnaire in our setting, and it was tested in a sample of people with characteristics similar to the study population, after which they were excluded from the study. The questionnaire consists of 17 items and is measured with scales that determine the following frequencies: 1 time a month or less = 0 point, 2 to 3 times a month = 1 point, 1 to 2 times a week = 2 points, 3 to 4 times a week = 3 points, and from 5 to more times a week = 4. The sum of the scores corresponding to 0–7 points is equal to a very low-fat intake or less than 25% of calories, 8–14 points are equal to an average fat intake, or between 30% and 35% of calories, 15–22 points are equal to a high-fat intake or greater than 35% of calories, and 23 or more points are equal to very-high-fat intake (40–50% of calories).

### 2.3. Measurement of BMI

The weight and height of the participants were self-reported. The BMI was used to evaluate the bodyweight category according to the criteria established by the Ministry of Health of Peru in the Technical Guide for the Anthropometric Nutritional Assessment of the Adult [27]. Participants were classified into four groups as follows: underweight (BMI < 18.5 kg/m^2^), normal weight (BMI 18.5 to < 25 kg/m^2^), overweight (BMI 25 to < 30 kg/m^2^), and obesity (BMI ≥ 30 kg/m^2^).

### 2.4. Statistical Analysis

Statistical analyzes were performed using the IBM SPSS statistical software package, version 24 (SPSS Inc., Chicago, IL, USA). Descriptive analysis of sociodemographic data, lifestyle, and dietary patterns was done using tables of absolute frequencies and percentages. Hypothesis testing was carried out for the comparison of proportions between men and women, and the categories of food addiction and fat intake were done using the Chi-square statistical test.

## 3. Results

Table 1 describes the sociodemographic data, lifestyle, dietary pattern, and BMI of the participants. Higher proportions of male and female participants aged 25 to 34 years were observed. Regarding the origin, it was found that the majority of the female participants came from the coastal and highlands of the country compared to the men; these differences are significant (*p* < 0.05). Regarding the degree of education, it was observed that the highest proportion of women (74.4%) had a professional title or a higher academic degree compared to men (65.4%); however, no significant differences were observed. Most of the men reported resting between 6 and 8 hours per night, compared to women (88% vs. 83.3%); however, there were no significant differences. On the other hand, significant differences were observed in terms of the frequency of physical activity between men and women, with a higher proportion of men who reported physical activity between 1 and 2 times a week, 3 and 4 times a week, and all days (90.1% vs. 72%, *p* < 0.001). However, regarding BMI, higher proportions of overweight male participants were observed compared to women; these differences are significant (*p* < 0.05). Finally, a higher proportion of women who had food addiction was observed compared to men; however, there were no significant differences (*p* > 0.05).

In Table 2, it is observed that the participants who did not have food addiction reported a slightly higher percentage of compliance with hours of adequate sleep compared to those with addiction (86.1% vs. 84.4%), but these differences were not significant (*p* < 0.05). Regarding BMI, more than half of the participants who were excess body weight were those with food addiction (54.1% vs. 38.6%); highly significant statistical differences were observed (*p* < 0.001). In relation to physical activity, those participants without food addiction reported activity more frequently compared to those with food addiction; significant differences were observed (*p*=0.017). Finally, regarding dietary patterns, a higher proportion of vegetarians without food addiction were observed; on the other hand, the highest percentage of nonvegetarians was found in the group that reported food addiction; these differences were significant (*p*=0.046).

In Table 3, it is observed that more than half of the participants who reported excess body weight were those who had a high intake compared to those who reported a low intake of saturated fat; these differences were significant (54.9% vs. 38.9, *p*=0.022). Regarding physical activity, it was observed that those participants who reported a low intake of saturated fat reported physical activity at an adequate frequency (3 to 4 times/week and every day) in a higher proportion, compared to those who reported a high saturated fat intake, and these differences are significant (*p*=0.047). Regarding the dietary pattern, a higher proportion of participants with a low intake of saturated fat was observed; on the other hand, the highest proportion of high intake of saturated fat was observed in nonvegetarians, with a statistically highly significant difference (*p* < 0.001). Finally, more than half of the participants who reported food addiction had a higher rate of high intake of saturated fat; on the other hand, the highest percentage of participants without food addiction was found in the group that reported a low intake of saturated fat (*p* < 0.001).

## 4. Discussion

In this online survey, we have evaluated anthropometric and lifestyle factors, food addiction, and saturated fat intake in Peruvian young adults.

In this study, it was found that more than half of the participants who had food addiction are excess body weight. This is consistent with the findings of other studies in which they found that food addiction is related to clinically relevant indicators, especially with a high BMI [28]. Similarly, another study found that BMI was significantly higher in food addicts compared to those who were not addicted to food [29, 30]. These findings support the proposal that food addiction is associated with the consumption of high-calorie foods, which, in turn, would increase the risk of obesity. In addition, they suggest that obesity characterized by addictive eating behaviors (other than eating disorders) may be particularly relevant in the evaluation of the causes and in the use of effective methods for the prevention and treatment of obesity [29]. However, it should be noted that other studies showed a nonlinear relationship between food addiction and BMI, with the highest prevalence among underweight and obese people [31], suggesting that food addiction may also reflect a distinct characteristic of inappropriate eating behavior that is not synonymous with excess body weight.

On the other hand, there were no significant differences between the proportions of men and women who had food addiction; however, a higher proportion of women who had food addiction was observed compared to men. However, other results suggest that addictive dietary eating behavior affects a considerable proportion of young, middle-aged women [13]. In fact, overweight or obese women have a significantly higher rate of food addiction compared to men [29]. This confirms the fact that eating disorders are more common among women than men [32]. However, larger studies in different populations are suggested to confirm the findings of the present study. On the other hand, in the present study, men were those who were excess body weight in a greater proportion compared to women. Other studies carried out in Peru showed similar results [33]. These findings are consistent with other studies in which overweight and obesity have been found to be more prevalent in women than in men [17]. Possible justifications include the fact that, in general, women are more concerned about their body image and weight control than men and that weight control is attributed to women than to men [34]. Likewise, women may have a higher concentration of leptin, an appetite-regulating hormone [35, 36]. However, there are discrepancies in the results of other studies [37], where it has been reported that women are more overweight and obese than men, showing that they have 2.2 times the risk of being excess body weight than men.

Moreover, it was observed that more than half of the participants who reported food addiction had a high intake of saturated fat; on the other hand, the highest percentage of participants without food addiction is found in the group that reported a low intake of saturated fat (*p* < 0.001). The current results are consistent with other findings in which food addiction and consumption of high-fat foods have been shown to be associated [10, 29]. In fact, people with food addiction report higher dietary fat intake than those without food addiction [17]. Similarly, in other studies, participants who had higher food addiction scores had a high percentage of saturated fat intake [11]; even when 116 overweight and obese people were examined, those who demonstrated food addictive behaviors were found to consume more grams of saturated and monounsaturated fat than nonobese subjects with statistically significant addiction [38]. On the other hand, studies show significant relationships between food addiction and a taste for fatty foods [39]. It is suggested that tasty foods rich in fat, salt, and sugar could be associated with an addictive diet [18]. The excessive consumption of these nutrients increases the chances of suffering from chronic noncommunicable diseases such as CVD, especially overweight and obesity [14, 19]. In addition, in this study, it was found that more than half of the participants who reported excess body weight were those who had a high intake of saturated fat as opposed to those who reported a low intake; these differences were significant (54.9% vs. 38.9, *p* < 0.05) (Table 3). Our findings support previous studies that observed positive associations between saturated fat intake and excess body weight [40]. This is due to the fact that fats are addictive due to their sensory characteristics and the high palatability generated by consuming them [41].

There is strong evidence from cross-sectional and longitudinal studies to support that unhealthy lifestyles, including diets high in saturated fat, high in calories, lack of sleep, and physical inactivity, are independent risk factors for overweight, obesity, and other chronic diseases such as CVD [42, 43]. In contrast, consuming a plant-based, low-fat diet with minimally processed, low-glycemic foods may contribute to the prevention of obesity and CVD [23]. Similarly, Mediterranean diets, in which they emphasize the consumption of fruits, vegetables, whole grains, olive oils, and particularly, fish, shellfish, and low-fat dairy products, can reduce cardiovascular events, such as myocardial infarction and stroke [21]. Furthermore, in terms of physical activity, studies show an obvious dose-response relationship between an increase in physical activity and a decrease in CVD and obesity risk [44]. In the present study, some lifestyle characteristics, such as hours of sleep, physical activity, and following a vegetarian dietary pattern, favor a lower probability of food addiction [13] and lower fat intake and, therefore, lower risk of overweight and obesity and CVD. It is important to emphasize that although several studies suggest that addictive eating behaviors are associated with obesity, it is unlikely that all people who are overweight and obese are addicted to food. Obesity is the result of a number of physiological and environmental factors, other than compulsive eating. A balanced and balanced diet, including an adequate intake of dietary fiber and an adequate supply of water, can contribute to proper control of body weight [45, 46].

## 5. Limitations

When evaluating the implications of this study, there are certain limitations that must be considered. In the first place, it is a cross-sectional study, so any interpretation regarding causality should be avoided; therefore, long-term prospective studies are suggested on the relationship between food addiction, saturated fat intake, and body weight gain over time. Second is the participants' self-report on weight and height, considering that people tend to underestimate their current weight and overestimate their height [47], which increases the probability of error in terms of BMI. Finally, we consider BMI as a limitation because it provides imperfect data on body composition, body fat distribution, and muscle mass [46]. In future studies, the use of alternative anthropometric measures to BMI is suggested to identify body composition with more precision. Despite these limitations, the current study suggests that the assessment of addictive-type eating behaviors may be useful in understanding the etiology of obesity. The results of the current study provide evidence on the need and importance of developing future programs.

## 6. Conclusion

In conclusion, in the current study, it appears that food addiction and saturated fat intake can lead to increased excess body weight. Additionally, participants who reported food addiction had also reported high saturated fat intake and less physical activity and were more likely to report fewer hours of sleep at night, which, consequently, may increase the risk of obesity and CVD. The findings of this study provide evidence on the importance and need of developing future nutritional education programs as part of efforts to prevent problems related to food addiction, high-calorie density food intake, obesity, and CVD.

## Figures and Tables

**Table 1:** Sociodemographic characteristics, lifestyle, diet, and BMI of the participants according to sex.

Variable	Women		Men		*χ* ^*2*^	*p* ^*∗*^
	*n*	%	*n*	%		
*Age (year)*						
18–24	44	21.7	25	13.1	5.074	0.166
25–34	112	55.2	115	60.2		
35–44	24	11.8	26	13.6		
>45	23	11.3	25	13.1		

*Origin*						
Coast	123	60.6	112	58.6	6.122	0.047^*∗∗*^
Sierra	67	33	53	27.7		
Jungle	13	6.4	26	13.6		

*Degree of instruction*						
Basic	17	8.4	27	14.1	5.590	0.133
Technical	35	17.2	39	20.4		
University	151	74.4	125	65.4		

*Hours of sleep*						
<6	22	10.8	18	9.4	2.923	0.232
6–8	169	83.3	168	88		
>8	12	5.9	5	2.6		

*Physical activity*						
Never	57	28.1	19	9.9	26.190	<0.001^*∗∗*^
1-2 times/week	86	42.4	110	57.6		
3-4 times/week	39	19.2	36	18.8		
5-6 times/week	15	7.4	11	5.8		
Diary	6	3	15	7.9		

*Diet regimen*						
Vegetarian	49	24.1	38	19.9	1.030	0.310
No-vegetarian	154	75.9	153	80.1		

*BMI (kg/m* ^2^)						
Underweight	3	1.5	4	2.1	7.493	0.048^*∗∗*^
Normal	122	60.1	92	48.2		
Overweight	50	24.6	70	36.6		
Obesity	28	13.8	25	13.1		

*Food addiction*						
Addiction	69	36.1	66	32.5	0.570	0.450
No addiction	122	63.9	137	67.5		

BMI, body mass index; ^*∗*^*p* value. The Chi-square test (*χ*2) was used to evaluate the degree of significance of the sociodemographic data, lifestyle, diet, BMI, and sex of the participants. *p* represents the probability that sex is associated with the aforementioned data. ^*∗∗*^Statistical significance.

**Table 2:** Hours of sleep, BMI, physical activity, diet, and food addiction.

	Addiction		No addiction		*X* ^2^	*p* ^*∗*^
	*n*	%	*n*	%		
*Hours of sleep*						
<6	17	12.6	23	8.9	2.103	0.349
6–8	114	84.4	223	86.1		
>8	4	3	13	5		

*BMI (kg/m* ^2^)						
Underweight	4	3	3	1.2	22.545	<0.001^*∗∗*^
Normal	58	43	156	60.2		
Overweight	41	30.4	79	30.5		
Obesity	32	23.7	21	8.1		

*Physical activity*						
Never	37	27.4	39	15.1	12.031	0.017^*∗∗*^
1-2 times/week	64	47.4	132	51.0		
3-4 times/week	19	14.1	56	21.6		
5-6 times/week	6	4.4	20	7.7		
Everyday	9	6.7	12	4.6		

*Diet regimen*						
Vegetarian	22	16.3	65	25.1	3.994	0.046^*∗∗*^
Nonvegetarian	113	83.7	194	74.9		

BMI, body mass index; ^*∗*^*p* value. The Chi-square test (*χ*2) was used to assess the degree of significance of the data on lifestyle, diet, BMI, and food addiction categories. *p* represents the probability that the food addiction categories are associated with the aforementioned data. ^*∗∗*^Statistical significance.

**Table 3:** Hours of sleep, BMI, physical activity, diet, and food addiction based on saturated fat intake categories.

	Low intake		High intake		*X* ^*2*^	*p* ^*∗*^
	n	%	n	%		
*Hours of sleep*						
<6	26	10.3	14	11.7	5.029	0.540
6–8	224	85.1	113	84.9		
>8	13	4.6	4	3.5		

*BMI (kg/m* ^2^)						
Underweight	6	2.5	1	0.6	19.388	0.022^*∗∗*^
Normal	156	58.7	58	44.5		
Overweight	76	28.6	44	33.5		
Obesity	25	10.3	28	21.4		

*Physical activity*						
Never	45	17.8	31	25.0	21.234	0.047^*∗∗*^
1-2 times/week	124	46.4	72	55.5		
3-4 times/week	60	22.9	15	10.3		
5-6 times/week	23	8.7	3	1.7		
Everyday	11	4.1	10	7.5		

*Diet regimen*						
Vegetarian	73	27.1	14	9.8	17.056	<0.001^*∗∗*^
Nonvegetarian	190	72.9	117	90.3		

*Food addiction*						
Addiction	56	22.7	79	62.6	67.424	<0.001^*∗∗*^
No addiction	207	77.2	52	37.4		

BMI, body mass index; ^*∗*^*p* value. The Chi-square (*χ*2) test was used to assess the significance of the data on lifestyle, diet, BMI, and categories of fat intake. *P* represents the probability that the saturated fat intake categories are associated with the aforementioned data. ^*∗∗*^Statistical significance.

## Data Availability

The datasets used and analyzed during the present study are available from the corresponding author on reasonable request.
